# Family Dogs’ Sleep Macrostructure Reflects Worsened Sleep Quality When Sleeping in the Absence of Their Owners: A Non-Invasive Polysomnography Study

**DOI:** 10.3390/ani15213182

**Published:** 2025-10-31

**Authors:** Luca Baranyai, Ivaylo Iotchev, Ferenc Gombos, Anna Kis

**Affiliations:** 1 “Momentum” Psychobiology Research Group, HUN-REN Institute of Cognitive Neuroscience and Psychology, Magyar Tudósok krt 2., 1117 Budapest, Hungary; brnyluc@gmail.com (L.B.);; 2 HUN-REN—ELTE—PPKE Adolescent Development Research Group, Mikszáth Kálmán tér 1-4., 1088 Budapest, Hungary; 3 Laboratory for Psychological Research, Pázmány Péter Catholic University, Mikszáth Kálmán tér 1-4., 1088 Budapest, Hungary; 4 ELTE—HUNREN NAP Comparative Ethology Research Group, Magyar Tudósok krt 2., 1117 Budapest, Hungary

**Keywords:** dog, sleep, attachment, EEG

## Abstract

**Simple Summary:**

Dog owners co-sleeping with their pet is a widely debated topic. Empirical data show that co-sleeping generally worsens owners’ sleep quality, although this is not reflected in their subjective reports. The effects on dogs’ sleep quality, however, have not been investigated. The present study provides the first evidence that dogs manifest different sleep patterns when sleeping in the presence versus in the absence of their owners. Sleeping together with the owner in an unfamiliar environment resulted in dogs’ shortened sleep onset time, increased sleep efficiency, and specifically spending more time in deep sleep, compared to sleeping with a friendly, unfamiliar human, who was not their owner. These findings align with the well-known fact that dogs show human-like attachment towards their owners.

**Abstract:**

Family dogs stand out with regard to their special (human-like) attachment behavior towards their owners. This dog–owner attachment bond, analogous to the human infant–mother relationship, has been extensively documented at the behavioral level. Capitalizing on the fully non-invasive polysomnography protocol, the current study compares family dogs’ sleep structure when sleeping in the company of their owners versus an experimenter (a friendly stranger human). Subjects (N = 9) participated in three recording sessions, each lasting 3 h. The first session served as an adaptation to the recording environment, while the second and third were the test sessions analyzed for the present paper. On these two occasions, dogs slept, in a counterbalanced order, once in the company of their owner, while on the other occasion they slept in the company of an experimenter, while the owner was outside the room. Polysomnography recordings were used to extract high-resolution information (in 20 s epochs) on the time dogs spend awake and in each of the sleep stages (drowsiness, non-REM, and REM). Our results show a robust difference between dogs’ sleep structure with and without the owner. In addition to an increased sleep latency and worsened sleep efficiency, dogs spent considerably less time in deep sleep (non-REM) when their owner was absent. These findings add to the increasing body of literature dealing with dog-to-owner attachment and provide unique physiological evidence for the phenomenon, complementing the widely reproduced behavioral data.

## 1. Introduction

The bond between dogs and humans is ancient [1] and displays key similarities with the mother–infant attachment [2,3] as conceptualized by Bowlby [4,5] and operationalized by Ainsworth et al. [6] in the “Strange Situation Test”. In the industrialized world, the intensity of this dog–human relationship seems to have increased explosively over the last decade [7,8] and with it, the anthropomorphizing of family dogs [9], their perceived role in the family [10,11], and the prevalence and quality of pet keeping [12]. Although similar dynamics might have played a role during initial domestication, it is not clear if the current surge in “pet love” is truly advantageous to both sides [7]. One currently controversial, but surprisingly common, pet-directed behavior is owners sharing the bed with their dog. Dog–owner co-sleeping has a relatively high prevalence [13], despite widely replicated adverse effects on the owners’ objective sleep quality [14,15] (see [16] for minor adverse effects only).

For the owner, co-sleeping is linked to more frequent movements, arousals, and awakenings [14,15]. Based on actigraphy data, the animals’ nighttime movements predict movement in the owners, which are, however, not subjectively detected or remembered [14]. How dog-owner co-sleeping affects the animals has not been thoroughly examined. So far, we only know that movements of the owner affect the animal less than vice versa [15]. Currently, canine sleep and the effects of owner presence have not been studied in a combined setting. However, each line of research suggests possible effects on sleep quality.

Owner presence is a strong mediator of dog behavior. Dogs consistently behave differently when tested with versus without their owners. Results mainly come from the Strange Situation test, used to measure dog-to-owner attachment, where it has been shown that dogs in the presence of their owner explore more actively, engage in more play and affiliative contact with a friendly stranger, and exhibit fewer signs of stress [3,17,18]. Differences in the way dogs behave with versus without their owners, however, do extend to other situations as well. For example, participating in a routine veterinary examination with the owner will make the dogs more relaxed and willing to enter the consultation room [19], as well as decrease the dog’s vocalization and alter stress signals such as yawning [20]. In an agonistic context, a dog’s heart rate response to a threatening stranger is buffered by the owner’s presence [2], and the dogs that are inherently aggressive will likely suppress these behaviors in the absence of their owners, decreasing predictive validity [21]. The presence and absence of the owners also explain most of the variation in dogs’ postures and behaviors during the Socially Acceptable Behavior Test [22]. Furthermore, dogs have been shown to obey commands better when their owner is attentive [23].

Because dogs show attachment towards their owners [3,24], it has been standard practice to have the dogs’ owners present in cognitive test situations [25]. This ensures that the observed behavioral responses are the dogs’ natural reactions. Dogs, however, also spend, day by day, a considerable proportion of their time without their owners. These inevitable separation periods can cause well-known stress symptoms [26,27], but the effects on dogs who do not show separation anxiety are less well-known. Spending, for example, sleeping time together with the owner versus in separation is a topic often debated as to its costs and benefits.

Since the advance of a non-invasive polysomnographic protocol for dogs over a decade ago [28], our group was able to study various aspects of sleep in human-kept dogs [29,30]. Dogs’ sleep structure can be reliably quantified [31] and is starting to be used in both basic research [32,33] as well as for veterinary purposes, such as identifying age-related cognitive dysfunction [34,35] or canine epilepsy [36,37]. In addition, frequency-band activity [32] and transients [38,39] associated with awake cognitive performance have also been identified in the animals’ sleep EEG (electroencephalography). The role of the owner, however, is only indirectly implied by studying sleep in dogs with varying levels of attachment to their caretaker [40]. Higher attachment scores were associated with more time spent in non-REM sleep, lower non-REM alpha power activity, and lower non-REM alpha–delta anti-correlation.

A positive effect of co-sleeping for dogs can be expected at least on the basis of the owner’s effect on the awake animal. The literature on mother–infant co-sleeping is also relevant, since infants and dogs each form attachment bonds with their caretakers, but theory and empirical findings align only partially. Evolutionary considerations by McKenna et al. [41] emphasize, above all, a positive effect of mother–infant co-sleeping for the infant. There has been some evidence for increased sleep efficiency in infants [42] when the parent sleeps in the same room, but direct contact is instead linked to more arousal and shorter deep sleep [43,44,45,46,47]. The higher level of transient arousals [43] and reduced deep sleep duration [47] have still been presented in a positive light, as possibly reducing the risk of SIDS (sudden infant death syndrome). This may be a valid interpretation concerning infants who are still vulnerable and physically endangered, but if co-sleeping is universally beneficial to the dependent part of a caretaker–receiver dyad [41], we may expect to only see increased sleep efficiency when the object of care is an adult animal. We therefore predict that dogs’ sleeping patterns will be affected differently from those of infants. Specifically, increased sleep efficiency (e.g., more time spent in non-REM sleep) is more likely given the stress-reducing effect of owners on the awake animal [3,17,18], which is also less vulnerable than a newborn and may thus not benefit from transient arousals.

The aim of the current study is to quantify, for the first time, sleep pattern differences in family dogs when sleeping in the presence versus in the absence of their attachment figures (owners). We thereby focus on the latency to falling asleep and the time spent in each sleep stage.

## 2. Materials and Methods

### 2.1. Subjects

Our subjects were N = 9 adult (>2 years of age) family dogs (4 mixed-breed dogs and 5 purebreds: Samoyed, Dachshund, Cocker Spaniel, Corgie, and Puli, with a mean age of 6.78 years, of which 4 are females). The owners accompanying the dogs were all adult females between 24 and 49 years of age. Dogs had been living with their owner for a duration of at least 1 year (N = 3 for between 1 and 2 years, N = 2 for between 3 and 4 years, N = 2 for between 5 and 6 years, N = 1 for between 7 and 8 years, and N = 1 for between 11 and 12 years duration). On a 7-point Likert scale to rate their relationship to the dog, N = 6 owners gave the maximum (7) rating, and N = 3 owners gave a rating of 6. All except one owner (N = 8) reported, at times, sleeping together with their dogs.

No previous training was necessary to take part in the present study. Each participant was assessed three times. The first session consisted of a three-hour afternoon recording in the sleep laboratory, with the dogs’ owners present. This was performed to adapt dogs to measurement conditions (adaptation data is not analyzed herein). The second and third occasions were each a 3-h-long afternoon recording with the owner or with an unfamiliar experimenter (without the owner) present during the measurement, in a counterbalanced order. For each subject, both the with-owner and without-owner sleep sessions were conducted following similarly active days and at the same time of day (with no more than a one-hour difference in start time). Accordingly, every dog underwent three recordings: an adaptation session, a with-owner session, and a without-owner session.

### 2.2. Procedure

Owners were first asked to fill out the Pet Attachment Questionnaire (PAQ) [48], a validated scale, which measures people’s attachment orientations in the domain of human-pet relations. For the current study, the Hungarian version of the questionnaire [49] was used, which, identically to the original questionnaire, measured Pet Avoidance and Pet Anxiety. The questionnaire consists of 26 items (statements) rated on a Likert scale from 1 (strongly disagree) to 7 (strongly agree). The items included statements such as “I prefer to keep a distance from my pet”, “Often my pet is a nuisance to me”, or “Being close to my pet is pleasant for me” (reverse scored). For the current study, the sum PAQ score was used, with higher scores indicating more negative feelings from the owner regarding pet avoidance and pet anxiety.

Dogs’ sleep was assessed using polysomnography (PSG), which simultaneously recorded neural activity (EEG), electrooculogram (EOG), electrocardiogram (ECG), respiration, and electromyography (EMG) [28]. In line with earlier work [50], scalp electrodes were positioned along the anteroposterior midline (Fz, Cz, and Pz) and over the left and right zygomatic arches (os zygomaticum; F7, F8). The Pz site served as the common reference, so Fz–Pz, Cz–Pz, F7–Pz, and F8–Pz were used as EEG channels, while the F7–F8 derivation provided the EOG signal [50]. The ground (G) electrode was placed over the left musculus temporalis. Gold-coated Ag|AgCl cup electrodes secured with EC2 Grass Electrode Cream (Grass Technologies, USA) were used. All scalp electrodes were positioned on the bone to reduce muscle and movement artifacts. ECG electrodes were attached bilaterally over the second rib, and EMG electrodes were placed bilaterally on the musculus iliocostalis dorsi. Respiratory activity was monitored with a thoracic respiratory belt.

Signals were acquired, prefiltered, amplified, and digitized using a SAM 25 R MicroMed Headbox (MicroMed Inc., Houston, TX, USA) at 1024 Hz per channel, with a hardware bandwidth of 0.5–256 Hz, sampling rate of 512 Hz, anti-alias filter at 1 kHz, and 12-bit resolution (±2 mV range). Additional second-order software filters at 0.016 Hz (high-pass) and 70 Hz (low-pass) were applied using System Plus Evolution software (MicroMed Inc., Houston, TX, USA; version 1.05.0001). Electrode impedances were maintained below 20 kΩ.

When arriving at the laboratory, each dog was given five to ten minutes to explore the room. After this familiarization period, owners helped the research team attach surface electrodes. Throughout electrode placement, all dogs received social reinforcement (e.g., petting and verbal praise), and some were additionally rewarded with treats. When owners remained in the room during recording, they were instructed to disable radio-frequency transmission on their phones, sit on the mattress on the floor (with the dog either beside them or on the mattress), and engage in a quiet activity such as reading, sleeping, or watching a movie on a laptop using earphones (with wireless transmission disabled). Once the dog was settled, either the experimenter or the owner left the room, and recording began.

### 2.3. Data Analysis

Sleep recordings were visually scored in accordance with standard criteria adapted for dogs [31]. The wakefulness stage was defined as the occurrence of fast activity in the EEG, high amplitude and frequency eye movements in the EOG, elevated muscle tone, and frequent movements (EMG channel). Drowsiness was defined as fast EEG activity in the EEG channels accompanied by decreased amplitude and frequency of eye movements in the EOG, lowered but observable muscle tone (EMG channel), and fairly regular respiration (Rsp channel). Non-REM sleep was defined as the occurrence of ≥15 μV delta (1–4 Hz) activity and/or sleep spindles (waves with 12–16 Hz frequency and ≥0.5 s duration) in the EEG, no or low amplitude eye movements in the EOG, relatively regular respiration (Rsp channel), and decreased muscle tone (EMG channel). REM sleep was defined as the occurrence of rapid eye movements in the EOG—also seen as artifacts in the EEG—fast EEG activity, muscular atonia (EMG channel), irregular respiration (Rsp channel), and heartbeat (ECG).

Data were processed and exported using software developed in our laboratory (Fercio’s EEG Plus, © Ferenc Gombos 2009–2025). Each recording was segmented into 20-s epochs, which were then manually classified as wakefulness, drowsiness, non-REM sleep, or REM sleep. This provided data for exporting the following macrostructural variables: sleep efficiency (% of recording time not awake—in drowsiness, non-REM, and REM sleep); sleep latency (minutes elapsed from recording onset until first drowsiness); time spent in drowsiness, non-REM, and REM (total duration in minutes).

Macrostructure variables recorded in measurements with versus without the owner were compared using paired samples *t*-tests (using Statistical Package for the Social Sciences, SPSS version 22; IBM Corp. 2013, Armonk, NY, USA).

Owner’s responses to the Pet Attachment Questionnaire items were summed up to a PAQ scale, resulting in a score between a minimum of 26 and to maximum of 182 (1–7 score on each of the 26 items). Difference scores were calculated for each dog regarding the macrostructure variables when sleeping with versus without their owners. These sleep macrostructure difference scores were plotted against the owners’ Pet Attachment values. Due to the limited sample size of the current study, these data were only used for an exploratory (descriptive) analysis, and no statistical models were run.

## 3. Results

**Figure 1:**
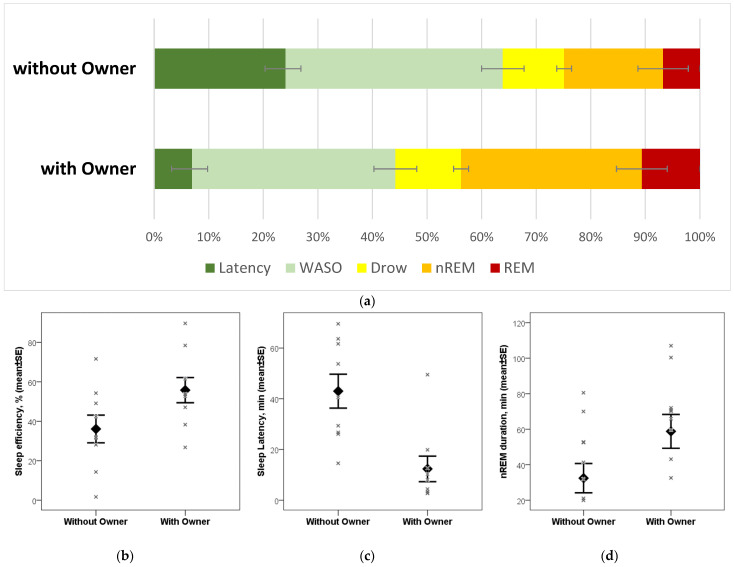
Sleep macrostructure differences between dogs’ sleep with versus without their owners: (**a**) mean ± SE values of each macrostructure variable on the two occasions. Latency: time elapsed from recording onset to first epoch classified as drowsiness; WASO: waking after sleep onset, summed duration of epochs classified as wake after the first instance of drowsiness; Drow: summed duration of epochs classified as drowsiness; non-REM: summed duration of epochs classified as non-REM; REM: summed duration of epochs classified as REM. (**b**–**d**) Significant differences in sleep efficiency, sleep latency, and non-REM duration, respectively; individual data points (marked with x-es) are shown in addition to mean ± SE values.

Subjects showed a markedly different sleep pattern when sleeping in the absence of their owner (Figure 1). With their owners present, dogs achieved significantly higher sleep efficiency (t_8_ = 3.463, *p* = 0.009), which stemmed from a shorter latency to fall asleep (t_8_ = 5.097, *p* = 0.001) and more time spent in non-REM sleep (t_8_ = 3.688, *p* = 0.006). There was no difference in the time spent in drowsiness (t_8_ = 0.226, *p* = 0.827) and in REM sleep (t_8_ = 1.178, *p* = 0.321).

**Figure 2:**
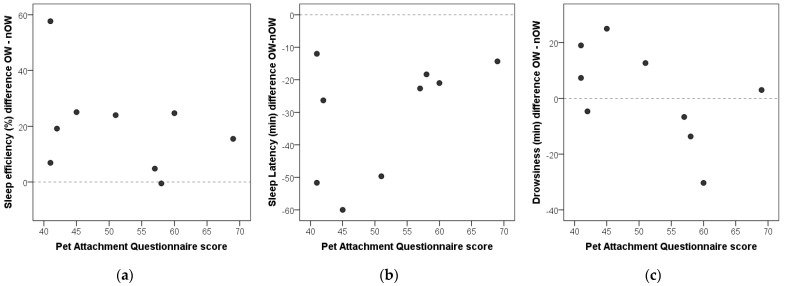
Individual variation between owners’ Pet Attachment (avoidance and anxiety) scores in relation to dogs’ sleep quality gain in terms of differences between owner-present (OW) versus no-owner-present (nOW) conditions for sleep efficiency (**a**), sleep latency (**b**), as well as drowsiness duration (**c**). Horizontal dotted lines at the 0 value depict no difference between owner and no-owner conditions.

The owner’s answers to the Pet Attachment Questionnaire varied between a sum score of 41 and 69 (on a scale from a minimum score of 26: no negative feelings related to the dog, to a score of 182: maximum possible negative feelings related to the dog). Due to the low sample size, no statistical correlation could be expected between the questionnaire scores and the dogs’ sleep difference in reaction to the owner’s presence or absence. Macrostructural variables where visual inspection of the data suggests possible tendencies worthy of future exploration are shown in Figure 2. In case of sleep efficiency, the dog who gained most from owner presence (+58%), was also the one whose owner reported the lowest indices of pet avoidance and anxiety (score: 41). Similarly, in terms of sleep latency gain, those three dogs that improved most in shortening sleep onset times due to owner presence (60 min, 51 min and 50 min, respectively) were among the ones with lowest PAQ scores (PAQ: 45, 41, 51). Regarding drowsiness duration, all except one of the dogs that spent more time in drowsiness with their owner present (difference values > 0) had below-median PAQ scores, while all except one of the dogs that spent more time in drowsiness with their owner absent (difference value < 0) had above-median PAQ scores.

## 4. Discussion

Results of the current study reveal marked differences in dogs’ sleep structure when measured in the presence versus in the absence of their owners. Although the findings are of a preliminary nature due to the limited sample size, the magnitude of the difference shows a significant decrease in sleep quality during the without-owner condition. This is in line with behavioral findings proving that dogs experience stress at different levels when being separated from their owners [51,52,53]. Furthermore, the dogs’ decreased sleep efficiency when measured without the owner aligns with some infant studies showing decreased sleep efficiency when the infant is separated from their attachment figure(s) [42,54]. The results, at the same time, do not align with other infant work, which instead reported increased arousal and decreased deep sleep in the presence of the parent [43,44,45,46,47]. Specifically, our findings show that the effect of co-sleeping on dogs most resembles what is observed when children sleep in the same room as their parents [42], but not what is seen when mother and infant embrace directly during sleep [43,44,45,46,47]. Of note, however, is that infant studies reporting deteriorative effects of co-sleeping (in the same bed) were recorded in the families’ home environments, whereas the current study on dogs was conducted in an unfamiliar laboratory. Overall, these findings add to previous work supporting the existence of a dog–human attachment bond, although future work must expand on how different levels of attachment, as well as different sleeping arrangements and environments, may affect co-sleeping.

A decrease in non-REM sleep was observed in the without-owner condition. The importance of non-REM sleep was also emphasized in earlier work on dogs [40], where several associations were reported between dogs’ sleep patterns and dog–human attachment measures. In another study [55], dogs were tested in a monotonous fetching task while their owners were watching, versus when they were present with their backs turned. It was found that the magnitude of difference in dogs’ individual behavior between the owner watching versus not-watching conditions (e.g., total number of trials during which the dog approached the toy to be fetched) was related to non-REM sleep EEG patterns (such as 2.5–4.0 Hz delta activity and 10.0–11.0 Hz alpha activity). Furthermore, based on results from [56], dogs’ non-REM sleep decreased, similarly to the present study, after a general negative (mildly stress-inducing) behavioral interaction. This latter study [56] included a short separation episode (when the dog was left alone in a room without the owner), and the duration dogs spent standing next to the door during separation was related at the individual level to the actual non-REM duration difference in that given dog between sleep following the positively versus negatively valenced pre-treatment.

Similar to the Strange Situation Test [57] used to quantify dog–human attachment, the current study also conducted measurements in an unfamiliar environment. The fact that subjects in this study were not sleeping at their homes might, at least partly, explain why no differences were found in the duration of REM sleep between the with and without owner conditions. It has been shown that when not at home, REM sleep following a first non-REM was less likely [58]; a floor effect relating to this phenomenon might mask any differences caused by the absence of the owner. A further possibility lies in the considerable individual variation inherent to dogs’ sleep (which is partly attributable to individual differences in age [59], sex [60], head shape [61], trait activity–impulsivity [62], etc.). The limited sample size of the current study did not allow for controlling these background variations, which might have masked some minor differences of smaller magnitude compared to the observed significant effects. As mentioned above, human research comparing the sleep of infants sharing a bed with parents versus sleeping on their own has been conducted in the families’ homes [43,44,45]; thus, direct parallels can hardly be drawn. While co-sleeping with parents in a home setting and under normal circumstances has generally been reported to worsen sleep quality [46,47], in the case of sleeping away from home and potentially accompanied by stressful events (such as being hospitalized) having a parent accommodated together with the child has been reported to reduce stress [63,64]—although sleep problems are universally reported in hospitalized settings [65].

Descriptive data were provided in the current study for potential associations between the magnitude of dogs’ sleep quality gain in relation to the owners’ pet attachment (avoidance and anxiety). Due to the low sample size, we did not conduct formal statistics on how the questionnaire and sleep data relate, but note that our preliminary results suggest that investigating the relationship between the dog–owner relationship and the owner’s effect on dogs’ sleep quality is a promising area for future research. Previous research [40] has shown that dogs’ attachment to the owner influences dogs’ sleep macrostructure in an unfamiliar environment (in the presence of their owner). However, despite available tools to measure owner attitudes towards pets [66,67], the interrelated nature of the dog–owner attachment is rarely considered as a bidirectional system. Human characteristics and attitudes were reported to influence the dog–human bond [68], and we also suggest that such measures should be taken into account when studying owner effects, further taking into account that the mother–infant attachment is also generally lacking in the human literature examining the effects of co-sleeping on sleep quality. Some studies, however, do account for maternal attitudes and report significant findings. Impairments in the mother-to-infant bond were, for example, linked to maternal insomnia [69]. On the other hand, maternal depression was linked to persistent co-sleeping even after controlling for parents’ attitudes toward preferred sleep arrangement [70]. Of note, in the current study, all except one of the participating dog owners involved reported regularly sleeping together with their dog. While the one subject who did not sleep together with the owner did not present as an outlier in any of the measured aspects, it would be important to examine in the future if owner presence in association with bidirectional measures of dog–human attachment inserts differential effects on the sleep patterns of dogs who are co-sleepers versus solitary sleepers.

Due to the current measurements having been conducted in an unfamiliar environment, results cannot be directly used to advise owners on home sleeping arrangements [71]. In addition to accounting for the dogs’ and owners’ regular sleeping arrangement, future studies should replicate these findings in the dogs’ home environments as well. However, the fact that significant improvement was detected when dogs were sleeping with their owners cannot be ignored. Sleep has vital functions in humans [72,73] as well as in dogs (and generally across mammals [30,74]). In particular, dogs were demonstrated to share the same processes that support offline processing and memory consolidation known from the human literature [32,38]. Changes in the associated EEG expression of these dynamics (e.g., increases in the intrinsic frequency of sleep spindles) are linked to similar cognitive problems (i.e., increased repetition errors [75,76]) in both species. Not getting proper sleep has serious medical consequences, spanning both physical and psychological disorders, like cardiovascular complications or depression, that are currently better explored in humans [77,78]. While in human sleep medicine, there are guidelines and definitions as to what counts as “proper sleep” [79], such measures are not in place for dogs, and it is thus even harder to evaluate the magnitude of the owners’ positive effect in the current study.

There are also several implications of the current findings for veterinary settings. Dogs experiencing sleep-related dysfunctions such as narcolepsy [80] or sleep-disordered breathing [81] will be referred to the clinic, and the current results provide guidelines for natural sleep pattern differences if diagnostic sleep measurements are performed in the presence versus absence of the owner. Additionally, non-invasive polysomnography can be a useful diagnostic tool even for neurological conditions not directly related to sleep, as recently suggested for epilepsy, for example [36,82]. Researchers working with special dog populations housed in non-home environments, such as shelter dogs [83], should also consider these results.

One key limitation of the current study, and polysomnography studies in general, is their labor-intensive nature, which allows only for limited sample sizes (e.g., N = 7 dogs included in the comparison of sleep following an active versus passive day in [28]). While these sample sizes are sufficient to find differences between groups, they are not suitable for investigating the effects of multiple confounding factors in the same study. For humans, alternative screening tools, such as questionnaires measuring sleep quality [84], are also in place to aid data collection from large samples. Despite offering less detailed sleep data, these can be very useful to target the interrelated effects of background variables. Recently, a sleep questionnaire has been developed and validated for dogs [85], which can be easily filled out by the owners. Future research could capitalize on this tool to complement polysomnography recordings in targeting the potentially joint effect of sleeping environment and individual variations in dog-owner attachment or other important measures.

## 5. Conclusions

In sum, the present findings provide evidence for the significant effect of owner presence on family dogs’ sleep structure in an unfamiliar environment. Specifically, in the absence of the owner, higher sleep latency as well as lower sleep efficiency, and less time spent in non-REM sleep were observed. Beyond advancing our understanding of dog–human attachment, these insights carry potential translational relevance for both veterinary practice and comparative research. Future studies conducted in naturalistic home environments, with larger and more diverse samples, will be essential to clarify the extent and mechanisms of these effects and to determine their practical implications for canine health and well-being.

## Data Availability

The raw data supporting the conclusions of this article are stored on the institutional NAS server and will be made available by the authors on request.
